# FGF21 facilitates autophagy in prostate cancer cells by inhibiting the PI3K–Akt–mTOR signaling pathway

**DOI:** 10.1038/s41419-021-03588-w

**Published:** 2021-03-22

**Authors:** Han Dai, Wenjing Hu, Lianying Zhang, Feiyu Jiang, Xiongmin Mao, Gangyi Yang, Ling Li

**Affiliations:** 1 The Key Laboratory of Laboratory Medical Diagnostics in the Ministry of Education and Department of Clinical Biochemistry, College of Laboratory Medicine, Chongqing Medical University, Chongqing, China; 2 Department of Endocrinology, The Second Affiliated Hospital, Chongqing Medical University, Chongqing, China; 3 Chongqing Prevention and Treatment Hospital for Occupational Diseases, Chongqing, China

**Keywords:** Prostate cancer, Tumour-suppressor proteins

## Abstract

Fibroblast growth factor 21 (FGF21) plays an important role in regulating glucose and lipid metabolism, but its role in cancer is less well-studied. We aimed to investigate the action of FGF21 in the development of prostate cancer (PCa). Herein, we found that FGF21 expression was markedly downregulated in PCa tissues and cell lines. FGF21 inhibited the proliferation and clone formation of LNCaP cells (a PCa cell line) and promoted apoptosis. FGF21 also inhibited PCa cell migration and invasiveness. The Gene Ontology and Kyoto Encyclopedia of Genes and Genomes analyses revealed that FGF21 was related to autophagy and the phosphatidylinositol 3-kinase–Akt kinase–mammalian target of rapamycin (PI3K–Akt–mTOR) pathway. Mechanistically, FGF21 promoted autophagy in LNCaP cells by inhibiting the PI3K–Akt–mTOR–70S6K pathway. In addition, FGF21 inhibited PCa tumorigenesis in vivo in nude mice. Altogether, our findings show that FGF21 inhibits PCa cell proliferation and promoted apoptosis in PCa cells through facilitated autophagy. Therefore, FGF21 might be a potential novel target in PCa therapy.

## Introduction

Prostate cancer (PCa) is one of the most common malignant tumors and is an important cause of cancer-related deaths in men. It also has the highest incidence rate among all types of malignant tumors in older men^1^. In recent decades, the incidence and mortality rate of PCa has increased in China^2^. Castration treatment can improve the prognosis of PCa; however, many patients will nevertheless progress to castration-resistant PCa^3^. Therefore, there is an urgent need for more effective PCa treatments.

The fibroblast growth factor (FGF) family is composed of 22 members, which can be divided into typical FGFs and hormone-like FGFs^4^. FGFs play an important role in cell growth and differentiation, angiogenesis, embryonic development, wound healing and repair, and metabolic regulation^5^. FGF21 is a member of the FGF ligand family and is mainly secreted by the liver. Like the gut-derived FGF15 and the bone-derived FGF23, FGF21 is involved in maintaining metabolic homeostasis^6^ (e.g., bile acid metabolism^7^, glucose and lipid metabolism, among others) and is related to the metabolic adaptation of the fasting state^8–10^. Under physiological conditions, the liver secretes a large amount of FGF21 protein into the circulation. In addition, FGF21 is also expressed in other tissues such as the pancreas, adipose tissue, muscle, and kidney under pathological and stress conditions^11^. Recent studies have also revealed that FGF21 is associated with the occurrence and development of tumors such as colon cancer^12^, thyroid cancer^11^, and liver cancer^13^. However, its role in PCa has not been reported to date. Moreover, although previous studies have shown that FGF19 and FGF23 can promote the occurrence and progression of PCa^14,15^, their underlying mechanisms are unclear. As FGF21 has a different molecular structure and tissue source from FGF19 and FGF23 and plays an essential role in metabolism in vivo, we speculate that FGF21 plays a distinct role in PCa occurrence and development. Here, we investigated the effects of FGF21 on PCa cell proliferation, migration, invasion, apoptosis, and autophagy, and its possible mechanisms.

## Materials and methods

### Tissue samples from patients

We obtained tissue specimens from 42 patients with PCa and 24 patients with benign prostatic hyperplasia (BPH) who had undergone surgery at the Second Affiliated Hospital, Chongqing Medical University (Chongqing, China), from March 2018 to March 2019. Two pathologists examined paraffin sections according to the World Health Organization (WHO) diagnostic criteria for the histological classification of PCa^16^. The PCa specimens were classified according to 2016 WHO Gleason score for PCa, one of the strongest clinical predictors of PCa progression and outcomes. The Gleason score stratifies PCa based on prostate-specific antigen, clinical stage, and biopsy score into low, intermediate, or high risk, where both 3 + 4 = 7 and 4 + 3 = 7 are considered the same within the intermediate-risk group. The specific groupings of the PCa tissues were as follows: 18 patients had Gleason score ≤7 and 24 patients had Gleason score >7^17^. The Chongqing Medical University Ethics Committee approved this study (2017-34), and informed consent was obtained from all participants.

### Gene Ontology (GO) and Kyoto Encyclopedia of Genes and Genomes (KEGG) enrichment analysis

Gene expression profiles from GSE33684 were downloaded from the Gene Expression Omnibus (GEO) online database (https://www.ncbi.nlm.nih.gov/geo/). GSE33684 is a microarray dataset from diet-induced obesity wild-type (WT) and FGF21 knockout (KO) mice. Differential expression genes (DEGs) were analyzed with the Limma software package (R3.6.1). Genes with |log2FC| > 1 and adjusted *p* value < 0.05 were identified as DEGs (i.e., their difference multiple was >2), indicating that their expression is closely related to FGF21. The lists of the GO and KEGG analyses were obtained through enrichment analysis. The molecular functions, biological processes, and cellular components of the genes were obtained. A *p* value of <0.05 was considered statistically significant^18^.

### Recombinant plasmid construction, cell culture, and treatments

LNCaP, 22Rv1, DU145, PC3, and RWPE-1 cells (American Type Culture Collection, Manassas, VA, USA) were maintained in RPMI-1640 medium. The PC3 cells were cultured in Dulbecco’s modified Eagle’s medium Nutrient Mixture F-12. All medium was supplemented with 10% fetal bovine serum and 1% penicillin/streptomycin.

Recombinant plasmids expressing FGF21 (pEGFP-FGF21, p-*FGF21*) and empty plasmids (pEGFP-N1, p-*N1*) were constructed and verified by *Xho*I and *Eco*RI digestion and DNA sequencing, as previously reported^19^. To investigate the effect of FGF21 on PCa cells, we transfected LNCaP cells with p-*FGF21* or p-*N1* for 72 h, and then incubated them with 5 or 25 mM glucose for 48 h. For mammalian target of rapamycin (mTOR) signaling pathway analysis, LNCaP cells that had been transfected with p-*FGF21* or p-*N1* were treated with or without MHY1485, an mTOR agonist, for 24 h.

### Cell viability and focus formation assay

Cell viability was examined using Cell Counting Kit-8 (CCK-8) according to the manufacturer’s instructions, as previously reported^20^. The viability of the control group was deemed 100% survival. For the focus formation experiment, LNCaP cells were transfected with p-*FGF21* or p-*N1*, and seeded into a 6-well plate. The cells were then cultured with glucose (5 or 25 mM) at 37 °C for 10 days. Crystal violet staining was used for counting the cell colonies. The experiments were repeated at least three times.

### Wound-healing and transwell migration experiments

LNCaP cells that had been transfected with p-*FGF21* or p-*N1* were seeded into 6-well culture plates. For the wound-healing experiment, a straight scratch was made in the cell monolayer with a pipette tip when the cells were 90% confluent. The cells were cultured in RPMI-1640 medium with 5 or 25 mM glucose for 24 h. The scratch was examined and photographed under a light microscope, and the cell-free area was quantified using the ImageJ software.

For the Transwell migration assay, the cells were seeded into the upper chamber of 24-well Transwell plates with 200 μL medium. Then, the lower chamber was filled with 600 μL 10% fetal bovine serum-supplemented medium, and the cells were cultured for 12 h. Subsequently, the cells on the upper surface of the chamber were removed using a cotton swab. The cells on the bottom surface were fixed in 4% paraformaldehyde (PFA) for 30 min and stained with 0.1% crystal violet for 10 min. The migrated cells were photographed under a microscope, and the images were analyzed using ImageJ.

### Flow cytometry analysis

LNCaP cells were seeded and cultured in 6-well plates for 48 h at 37 °C, and then transfected with p-*FGF21* or p-*N1* for 72 h. The cells were collected and washed in ice-cold phosphate-buffered saline, then stained with annexin V-allophycocyanin (APC-A) and 4′, 6-diamidino-2-phenylindole (DAPI) PB450-A from an Apoptosis Detection Kit (BD Biosciences Inc., San Jose, CA, USA) according to the manufacturer’s instructions. For determining the cell cycle phase, the cells were fixed in 75% ice-cold ethanol overnight and then treated with 1 mg/mL RNase (Sigma-Aldrich) for 10 min at 37 °C. DNA was stained with propidium iodide (15 mg/mL) for 20 min at 4 °C in the dark. Cell cycle profiles and apoptosis were analyzed by flow cytometry (FACS Vantage SE, BD, Franklin Lakes, NJ, USA).

### Tumorigenicity experiments in nude mice

For the tumorigenicity experiments, we used 5-week-old male BALB/c nude mice from Huafukang Biotechnology Co., Ltd (Beijing, China). LNCaP cells infected with p-*FGF21* or p-*N1* (5 × 10^6^ cells, 200 μL) were injected subcutaneously into the dorsal thighs of the mice. Tumor growth was observed for 3 weeks, and the tumor volume was calculated as *V* = length × width^2^ × 0.5. After 3 weeks, the mice were sacrificed, and the tumor tissue was collected and frozen in liquid nitrogen for subsequent analysis. All animal experiments were supported by the Chongqing Medical University Animal Experimentation Ethics Committee (2018-048).

### Hematoxylin–eosin (H&E) and immunohistochemistry staining

H&E staining was performed according to routine protocols^21^. For immunohistochemistry (IHC) staining, tumor tissues from the patients with PCa and BPH were fixed in 4% PFA at 4 °C overnight, embedded in paraffin, and sectioned to 7 μm thickness. IHC was performed using an antibody against FGF21 (1:250, ab171941, Abcam, Cambridge, UK).

### Immunofluorescence assays

LNCaP cells were transfected with p-*FGF21* or p-*N1* and cultured for 72 h. The cells were incubated with 0.1% Triton X-100 at room temperature (RT) for 15 min, and then blocked with 5% goat serum at 37 °C for 30 min. Next, the cells were incubated with primary antibody against LC3B (1:200, NB100-2220, Novus Biologicals, Colorado, USA) at 4 °C overnight, and then with the secondary antibody at RT for 1 h, before being stained with DAPI for 1 min.

### Transmission electron microscopy (TEM)

LNCaP cells were fixed in 2.5% glutaraldehyde for 1 h and washed twice with 0.1 mol/L sodium cacodylate. The cells were then fixed in 2% osmium tetroxide for 2 h, washed with distilled water, and stained with 0.5% uranyl acetate. Next, the cells were dehydrated in ethanol, rinsed in propylene oxide, and embedded in 1:1 propylene oxide:Spurr’s resin for 24 h. Ultrathin sections (80 nm) were cut with an ultra-microtome. Images were obtained under a Hitachi HT-7700 TEM (Hitachi, Tokyo, Japan).

### Quantitative RT-PCR (qRT-PCR) and western blotting

qRT-PCR was performed as previously described^22^. The primer pairs are listed in Table S1. Western blot analysis was performed as described previously^23^. The primary antibodies included anti-LC3 (1:1000, NB100-2220, Novus Biologicals, Colorado, USA), anti-FGF21 (1:1000, ab171941, Abcam, Cambridge, UK), anti-P62 (1:1000, ab56416, Abcam, Cambridge, UK), anti-Ki-67 (1:1000, ab16667, Abcam, Cambridge, UK), anti-LAMP2 (1:500, ab13524, Abcam, Cambridge, UK), anti-BCL-2 (1:1000, #15071T, Cell Signaling Technology, Massachusetts, USA), anti-Akt kinase (Akt; 1:1000, #9272s, Cell Signaling Technology, Massachusetts, USA), phospho-Akt (Ser 473; 1:1000, #4060s, Cell Signaling Technology, Massachusetts, USA), anti-mTOR (1:1000, #2972s, Cell Signaling Technology, Massachusetts, USA), phospho-mTOR (1:1000, #2971s, Cell Signaling Technology, Massachusetts, USA), anti-p70S6K (1:1000, #2708s, Cell Signaling Technology, Massachusetts, USA), phospho-p70S6K (Thr389; 1:1000, #9205s, Cell Signaling Technology, Massachusetts, USA), and anti-β-actin (1:1000, TA-09, ZSGB-BIO, Beijing, China).

### Statistical analysis

Data were expressed as the mean ± SD or SE. GraphPad Prism software (version 6.0) was used to draw bar and line charts. The differences between the groups were assessed by a two-way ANOVA with a post hoc test. The two groups were compared by the Student’s *t* test. Statistical analyses were performed using SPSS software (version 19.0, Chicago, IL, USA). A *p* value < 0.05 was considered statistically significant.

## Results

### Expression of FGF21 in PCa tissues and cell lines

**Fig. 1:**
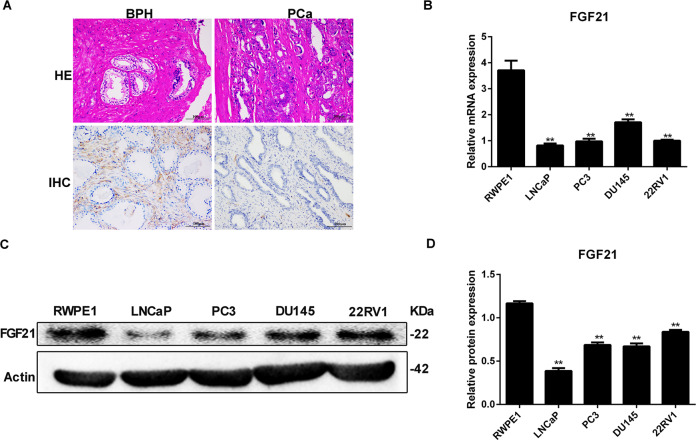
Expression of FGF21 in prostate cancer tissues and cell lines. **A** H&E and FGF21 IHC staining in tissues from PCa and BPH. **B**, **C** FGF21 mRNA (**B**) and protein (**C**) expression in different PCa cell lines. **D** Quantitative analysis of FGF21 protein expression. RWPE-1, prostate epithelial cells. Data were expressed as the mean ± SD. **p < 0.01 vs. RWPE-1.

We performed H&E and FGF21 IHC staining of tissues from 42 patients with PCa and 24 patients with BPH. The patients with PCa had lower FGF21 expression levels than the patients with BPH (Fig. 1A).

Further, we measured FGF21 mRNA and protein expression in prostate epithelial cells (RWPE-1) and PCa cell lines (i.e., LNCaP, PC3, DU145, 22Rv1) using RT-PCR and western blotting. All PCa cell lines had significantly lower FGF21 mRNA expression than the RWPE-1 cells (Fig. 1B). Moreover, all PCa cell lines had significantly lower FGF21 protein expression than the RWPE-1 cells (Fig. 1C, D). As LNCaP cells showed the lowest FGF21 protein expression levels, we selected that cell line for further study.

### Association of FGF21 with the pathological features of patients with PCa

We analyzed the relationship between FGF21 expression and the clinical characteristics of the patients with PCa. Table S2 shows that FGF21 expression was closely related to the pathological stage and Gleason score, suggesting that it might be involved in the development of PCa.

### FGF21 inhibits LNCaP cell proliferation and clone formation and promotes apoptosis

**Fig. 2:**
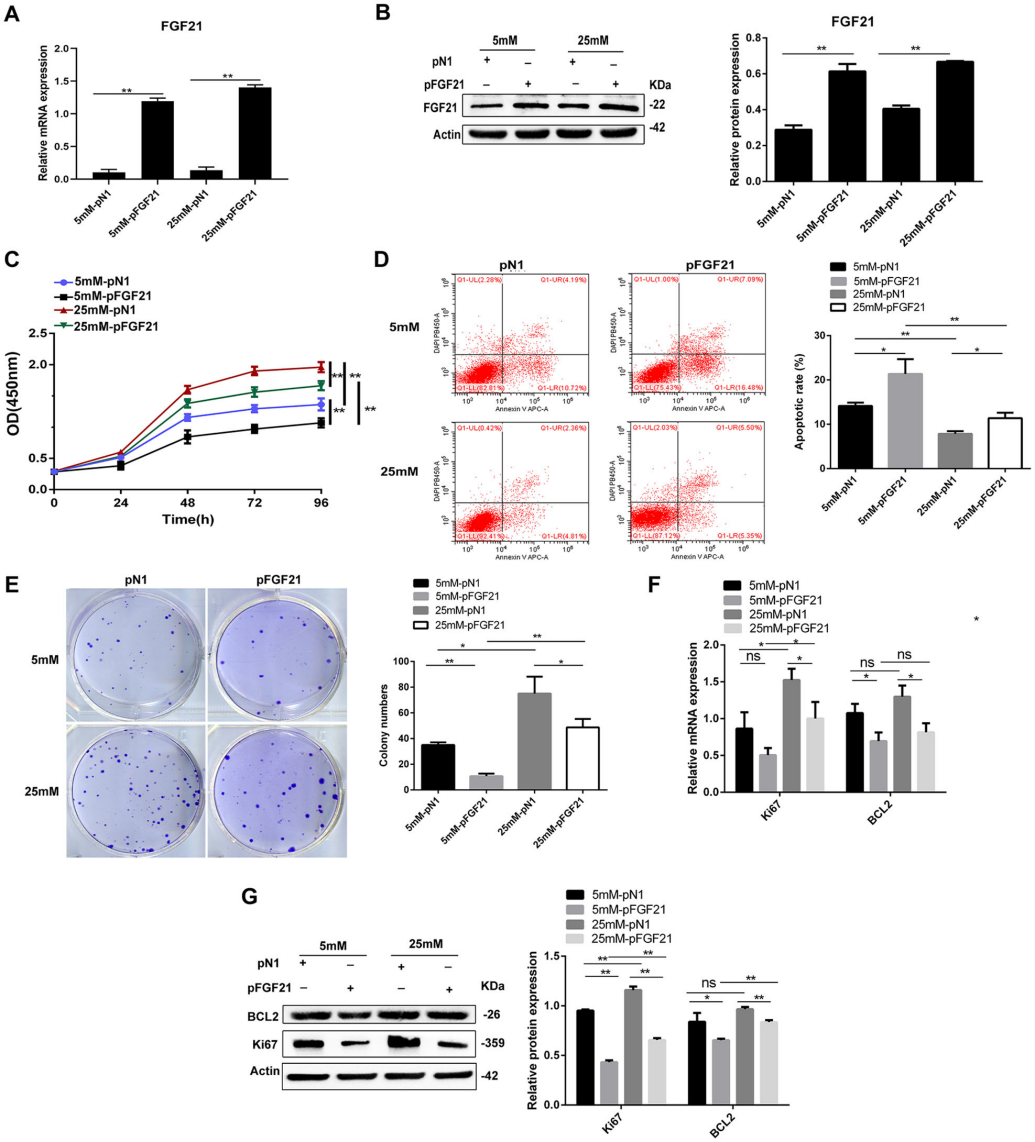
FGF21 inhibits cell proliferation and clone formation and promotes cell apoptosis in LNCaP cells. LNCaP cells were transfected with p-*FGF21* or p-*N1* for 72 h and incubated with 5 or 25 mM glucose for 48 h. **A** FGF21 mRNA expression. **B** FGF21 protein expression. **C** Effect of FGF21 overexpression on cell proliferation by CCK-8 assay. **D** Effect of FGF21 overexpression on cell apoptosis measured by flow cytometry assay. **E** Effect of FGF21 overexpression on the proliferation ability by colony formation assay. **F**, **G** FGF21 overexpression downregulated BCL-2 and Ki-67 mRNA (**F**) and protein (**G**) expression. Data were expressed as the mean ± SD. *p < 0.05, **p < 0.01, and n.s. not significant.

To investigate the effects of FGF21 on cell proliferation and clone formation in vitro, we transfected LNCaP cells with p-*FGF21* or p-*N1*. Further, as recent studies have shown that high glucose levels promote tumor cell proliferation and migration^24^, we cultured the cells in low (5 mM) and high (25 mM) levels of glucose. As expected, FGF21 expression was significantly increased at both mRNA and protein levels in the p-*FGF21*-transfected LNCaP cells (Fig. 2A, B).

Next, we used CCK-8 to detect the effect of FGF21 on LNCaP cell proliferation under different glucose concentrations. The proliferative ability of LNCaP cells increased significantly in hyperglycemic conditions (i.e., 25 mM glucose) compared with low-glucose (5 mM glucose) culture. However, the proliferative ability of the p-*FGF21* cells was significantly decreased (Fig. 2C).

We also examined the impact of FGF21 on LNCaP cell apoptosis by flow cytometry. LNCaP cells in hyperglycemic conditions had decreased levels of apoptosis than the cells in hypoglycemic conditions. FGF21 overexpression significantly increased LNCaP cell apoptosis under both hypoglycemic and hyperglycemic conditions (Fig. 2D). Furthermore, FGF21 overexpression significantly inhibited the hyperglycemia-induced clonal formation in the LNCaP cells (Fig. 2E). qRT-PCR and western blotting showed that FGF21 overexpression significantly inhibited the mRNA and protein expression of the cell proliferation and anti-apoptosis markers, including Ki-67 and BCL-2 (Fig. 2F, G). These data indicate that FGF21 inhibits LNCaP cell proliferation and clone formation, and promotes apoptosis, especially under high-glucose conditions.

### FGF21 inhibits LNCaP cell migration and invasiveness

**Fig. 3:**
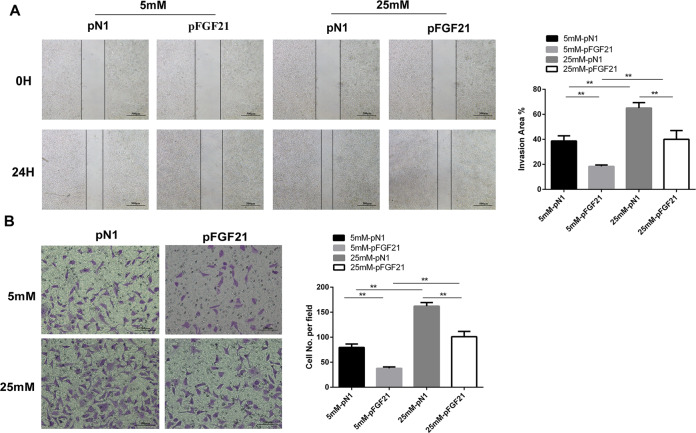
FGF21 suppresses cell migration and invasiveness in vitro. LNCaP cells were transfected with p-*FGF21* or p-*N1* for 72 h and incubated with 5 or 25 mM glucose for 48 h. **A** The mobility of LNCaP cells was measured by a wound-healing assay. **B** Cell invasion was measured by a Transwell migration assay. Data were expressed as the mean ± SD. **p < 0.01.

We observed the impact of FGF21 on LNCaP cell migration and invasiveness with wound-healing and Transwell migration assays. The wound-healing assay showed LNCaP cell mobility was increased under hyperglycemic conditions (Fig. 3A). However, compared with p-*N1* treatment, p-*FGF21* treatment led to a significant reduction in the mobility of LNCaP cells cultured with 25 mM glucose (Fig. 3A). In the Transwell migration experiments, LNCaP cells exposed to hyperglycemic conditions had significantly increased numbers of invaded cells. However, p-*FGF21* transfection reduced the number of invaded cells under both high- and low-glucose conditions (Fig. 3B). These results suggest that p-*FGF21* inhibits LNCaP cell migration and invasiveness.

### Identification of the genes and signaling pathways related to FGF21-mediated apoptosis

**Fig. 4:**
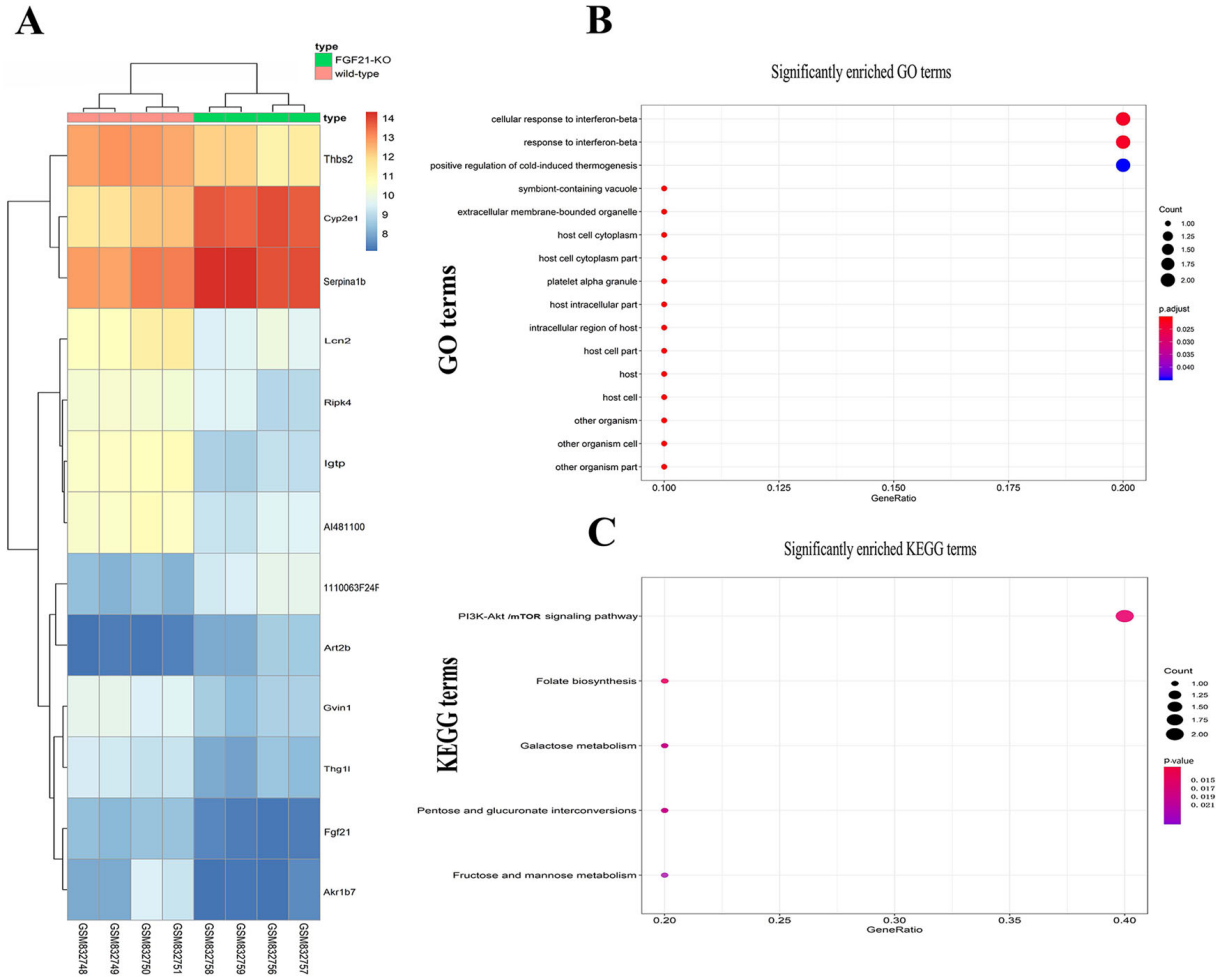
Identification of genes and signal pathways related to FGF21. **A** The heat maps for DEGs. Red and blue colors indicate relative expression above or below the average. **B** Functional analysis of DEGs. **C** KEGG pathway analysis for 13 DEGs. More red color indicates the smaller *p* values. DEG differentially expressed gene.

To determine the underlying mechanism of FGF21-mediated apoptosis, we analyzed gene expression profiles and candidate signal pathways from a GEO dataset (GSE33684). GO analysis showed that among 13 DEGs, nine were upregulated, and four were downregulated (Fig. 4A). These genes are involved in the cellular response to interferon-beta 2 (IFN-β2), response to IFN, and positive regulation of cold-induced thermogenesis, among others (Fig. 4B and Table S3). Of these 13 DEGs, thrombospondin-2 (*THBS2*) was related to B cell lymphoma 2 (BCL-2) (an apoptotic protein), and autophagy^25^.

KEGG pathway analysis showed that FGF21 is mainly related to the following signaling pathways: the phosphatidylinositol 3-kinase (PI3K)/Akt/mTOR, folate biosynthesis, and fructose and mannose metabolism (Fig. 4C and Table S4). Previous studies have found that the PI3K–Akt–mTOR signaling pathway is associated with autophagy^26^. Therefore, these results suggest that FGF21 is related to the PI3K–Akt–mTOR signaling pathway and autophagy.

### FGF21 promotes autophagy in LNCaP cells

**Fig. 5:**
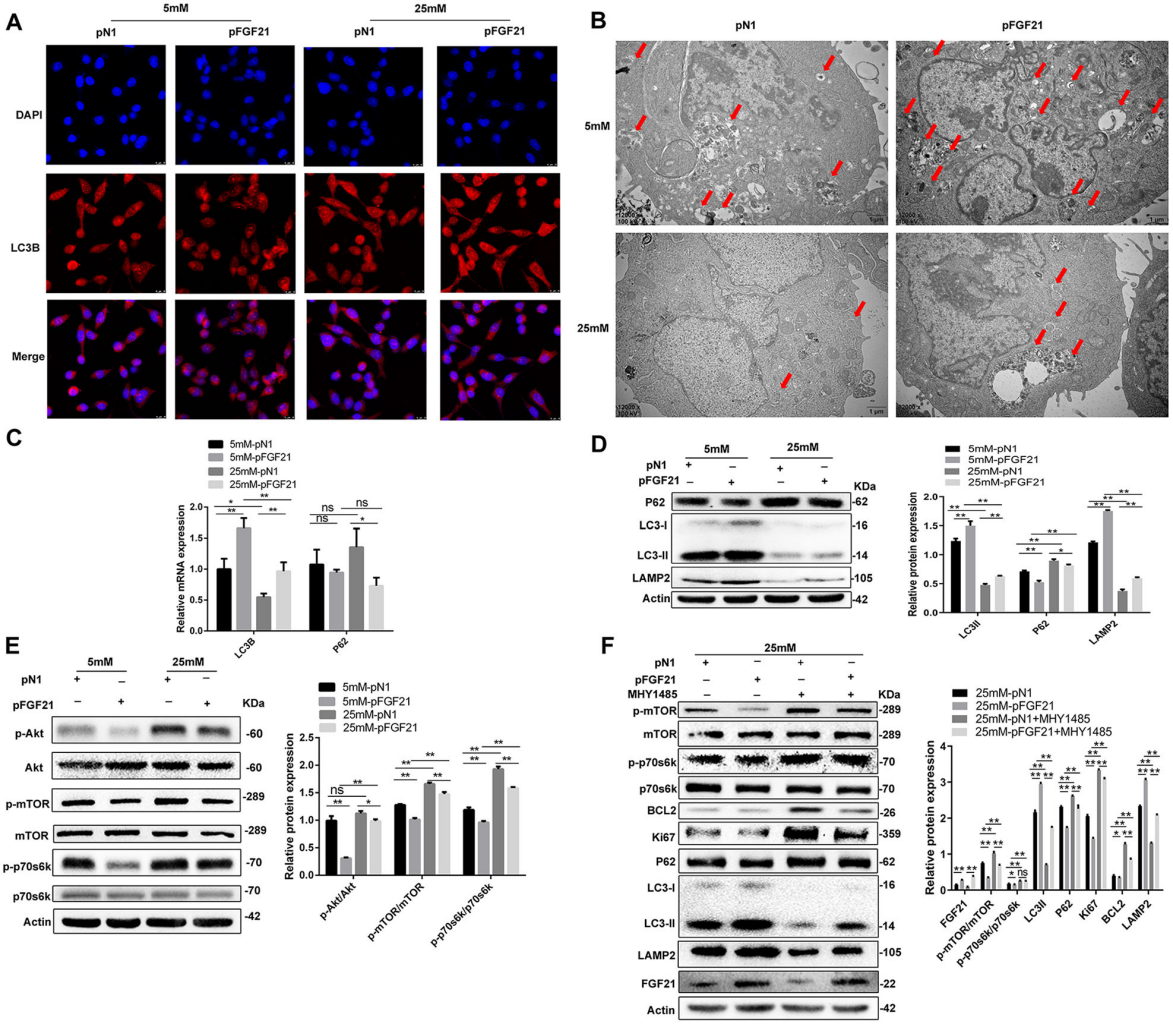
FGF21 promotes autophagy in LNCaP cells by inhibiting PI3K/Akt/mTOR signaling pathway. LNCaP cells were transfected with p-*FGF21* or p-*N1* for 72 h and incubated with 5 or 25 mM glucose for 48 h. In **F**, LNCaP cells were treated with or without MHY1485. **A** Immunofluorescence staining for LC3B expression in LNCaP cells (×20 magnification). **B** Transmission electron microscopy (TEM) pictures for detecting the autophagosome. Red arrows: autophagosome (×12,000 magnification). **C** LC3B and p62 mRNA expression. **D** The protein expression of LC3-I/II, p62, and LAMP2, and quantification of the protein levels. **E** The protein expression of p-Akt, p-mTOR, and p-p70S6K, and quantification of the protein levels. **F** The protein expression of p-mTOR, p-p70S6K, BCL-2, Ki-67, p62, LAMP2, and LC3-I/II in MHY1485-treated LNCaP cells. Data were expressed as the mean ± SD. *p < 0.05, **p < 0.01, and n.s. not significant.

Previous studies have shown that the activation of autophagy inhibits cell proliferation^27^, and our GO and KEGG pathway analyses showed that FGF21 is related to autophagy. Therefore, we studied the effect of FGF21 on autophagy in vitro to investigate the mechanisms underlying the FGF21-mediated inhibition of cell proliferation. First, we examined the expression of LC3B (a central protein in the autophagy pathway) in LNCaP cells via immunofluorescence staining. Figure 5A shows that there were reduced levels of LC3B in LNCaP cells under hyperglycemic conditions relative to hypoglycemic conditions. However, in the p-*FGF21* cells, immunofluorescence staining of LC3B was increased (Fig. 5A). Second, TEM showed that there were fewer autophagosomes in LNCaP cells under hyperglycemic conditions than that in hypoglycemic conditions. The number of autophagosomes in the p-*FGF21* cells was increased under both low- and high-glucose conditions (Fig. 5B).

To confirm the effect of FGF21 on autophagy, we used qRT-PCR and western blotting to examine the expression of LC3-I/II, p62, and LAMP2, three autophagy markers, in LNCaP cells. The overexpression of FGF21 increased LC3-II and LAMP2 expression and decreased p62 expression at mRNA and protein levels in the LNCaP cells (Fig. 5C, D). These data indicate that FGF21 increases the conversion of LC3-I to LC3-II and promotes autophagy in these cells.

### FGF21 inhibits PI3K–Akt–mTOR signaling in LNCaP cells

KEGG pathway analysis showed that FGF21 is related to PI3K–Akt–mTOR signaling. Therefore, we investigated the effects of FGF21 on the PI3K–Akt–mTOR pathway in LNCaP cells to determine how FGF21 can regulate autophagy in PCa. Under hyperglycemic conditions, the phosphorylation of Akt, mTOR, and p70S6K (a downstream target activated by mTOR complex 1 [mTORC1]) was increased in LNCaP cells (Fig. 5E). However, in the FGF21-overexpressing LNCaP cells, the effects of high glucose on Akt, mTOR, and p70S6K phosphorylation levels were reduced (Fig. 5E).

To confirm that FGF21-mediated autophagy is driven by mTOR signaling, we transfected LNCaP cells with p-*N1* or p-*FGF21* in the presence or absence of MHY1485, an mTORC1 agonist. mTOR activation blocked the FGF21-induced reduction of mTOR and p70S6K phosphorylation, and the downregulation of BCL-2, Ki-67, and p62 in the cells (Fig. 5F). In addition, MHY1485 inhibited the increased LAMP2 expression induced by FGF21 and attenuated the FGF21-induced conversion of LC3-I to LC3-II (Fig. 5F). These data indicate that FGF21 inhibits PI3K–Akt–mTOR signaling and facilitates autophagy.

### FGF21 inhibits PCa tumorigenesis in vivo

**Fig. 6:**
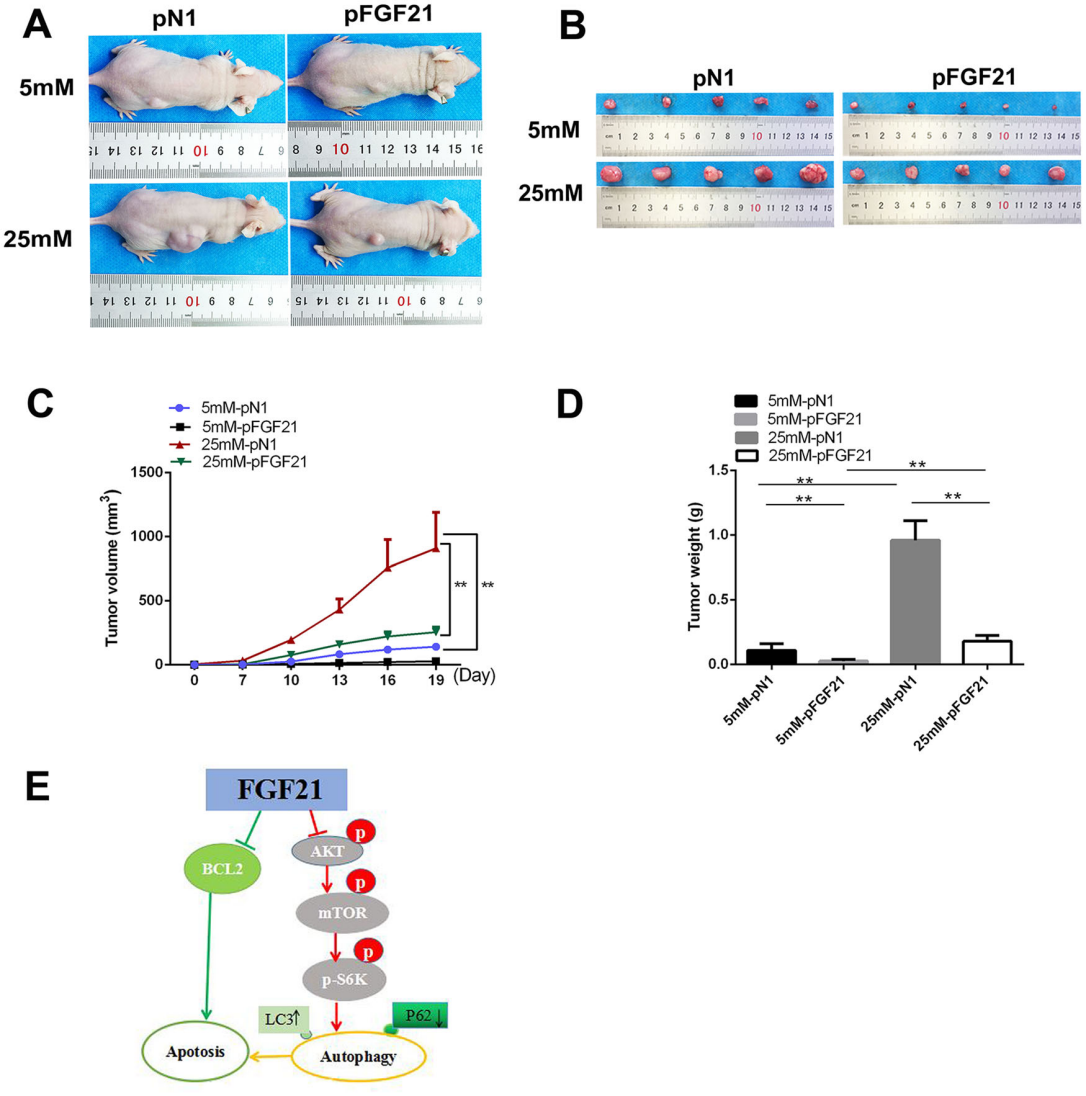
FGF21 overexpression represses tumor growth in vivo. FGF21-overexpressing LNCaP cells were subcutaneously injected in nude mice and representative tumor tissues were observed for 3 weeks. **A** The overall image of the tumor in nude mice. **B** Macroscopic pictures of tumors. **C** Tumor volumes in different groups. **D** The weights of tumors in different groups. **E** Schematic representation of the proposed mechanism of FGF21 in PCa cells. Data were expressed as the mean ± SD. **p < 0.01.

We studied the impact of FGF21 on tumorigenesis in vivo using PCa xenograft models. Nude mice were subcutaneously implanted with p-*N1-* or p-*FGF21*-transfected LNCaP cells (at concentrations of 5 and 25 mM d-glucose). The mice injected with high glucose-treated LNCaP cells had greater tumor volumes than the mice injected with the low glucose-treated LNCaP cells (Figure 6A–C). In addition, under both high- or low-glucose conditions 19 days after transplantation, the mice injected with p-*FGF21* cells had smaller tumor volumes than the mice implanted with p-*N1* cells (Fig. 6A, B). Furthermore, the tumors from the p-*FGF21* cells were markedly smaller and lighter than those from p-*N1* cells (Fig. 6C, D). When the transplanted cells were cultured with 25 mM d-glucose, the inhibitory effect of p-*FGF2*1 on the tumor was more obvious (Fig. 6C, D). These data indicate that FGF21 overexpression inhibits the growth of PCa in vivo.

## Discussion

Although previous studies have shown that FGFs derived from tumor cells or stromal cells induce tumor progression in several cancer types^28–31^, their precise role remains unclear. A recent study revealed that FGF21 mediates invasion and metabolic disorders in thyroid cancer and that elevated serum FGF21 levels in patients with thyroid cancer may be a marker of tumor progression^12^. However, the role of FGF21 in the occurrence and development of PCa is unknown.

Here, we demonstrate that: (1) FGF21 expression is decreased in both PCa tissues and cell lines; (2) FGF21 inhibits the proliferation, clone formation, migration, and invasiveness of LNCaP cells (a PCa cell line) and promotes their apoptosis; (3) FGF21 overexpression attenuates high glucose-induced LNCaP cell proliferation and apoptosis; (4) FGF21 is related to autophagy and the PI3K–Akt–mTOR pathway; and (4) FGF21 increases autophagy by inhibiting the PI3K–Akt–mTOR signaling pathway.

FGF21 is a cytokine derived from metabolism-related tissues (e.g., the liver, muscle, adipose tissue) and plays an important role in regulating glucose and lipid metabolism and metabolic adaptation in vivo. As tumor cells are metabolically active and vulnerable to nutrient starvation^32^, FGF21 may affect their phenotype. Indeed, in the current study, we found that FGF21 inhibited LNCaP cell proliferation, clone formation, migration, and invasiveness, and promoted apoptosis. Our results are similar to two recent in vivo studies that found hepatic FGF21 expression was activated, and serum FGF21 levels were increased in response to hepatocarcinogenesis, breast cancer, primary renal tumors, and papillary thyroid cancer^12,33–35^. These were mostly cross-sectional, population-based studies examining phenotypic characteristics, and without in-depth molecular biology studies. In those studies, the increased circulating levels of FGF21 observed in the patients with tumor might have been due to the increased synthesis and release of FGF21 in tumor cells, or the metabolic disorder of the microenvironment caused by tumor stress^35^. Therefore, in the present study, although we did not measure the circulating FGF21 levels, we believe that circulating FGF21 levels are increased in patients with PCa.

The tumor microenvironment plays an important role in tumor cell growth. High glucose promotes tumor cell proliferation and migration^25^. Given the role of FGF21 in glucose metabolism, we investigated its effects on LNCaP cell proliferation and migration under high-glucose conditions. We found that FGF21 overexpression significantly inhibited the proliferation and migration of LNCaP cells induced by high glucose. Herein, the effect of FGF21 on glucose metabolism in LNCaP cells might be similar to that in previous reports of obese and type 2 diabetes mellitus populations and animals. Those studies reported that FGF21 reduced blood glucose, blood lipid, and insulin levels, and improved glucose metabolism and insulin sensitivity^36–41^. Therefore, our data suggest that FGF21 affects glucose metabolism in LNCaP cells, for example, it may inhibit the utilization of glucose in LNCaP cells and thereby inhibits their proliferation. In addition, we found that the tumor volume in mice implanted with high glucose-treated LNCaP cells was greater than that in mice implanted with low glucose-treated cells. Indeed, growing evidence has shown that cells exposed to a hyperglycemic environment can affect the change in gene expression through epigenetic modifications, and this effect is sustained even if there is no hyperglycemia environment, which is called hyperglycemic memory^42,43^. The epigenetic modification of genes is very important for PCa progress^44,45^. Therefore, we thus believe that the epigenetic modification of gene expression in the LNCaP cells may be the reason for their faster growth in the nude mice.

Autophagy is a cellular process that degrades intracellular organelles and proteins to maintain cellular homeostasis^46,47^. Autophagy-related cell death is considered an important mechanism of cell death^48,49^. It was recently proved that autophagy can regulate glucose and lipid metabolism in tissues and cells^50^ and affects tumorigenesis^51^. Indeed, the activation or inhibition of autophagy plays an essential role in tumor growth^52,53^. Here, we observed that FGF21 overexpression increased LC3-I conversion into LC3-II, and the expression of LAMP2 protein (a well-known marker of autophagosome formation)^54^ reduced p62 levels and increased autophagosome formation in LNCaP cells, which suggests that FGF21 facilitates autophagy. Autophagy has also recently been found to be vital for inhibiting proliferation and promoting apoptosis in tumor cells^55,56^. Therefore, we speculate that FGF21 may inhibit the proliferation of PCa cells and trigger apoptosis through autophagy.

An increasing number of reports show that the PI3k–Akt signaling pathway plays a major role in tumor cell apoptosis and proliferation^57^. In addition, mTOR, a downstream mediator of the PI3K–Akt pathway, is related to the regulation of autophagy^58^. Other studies have also shown that the PI3K/Akt/mTOR signaling pathway exerts an antiapoptotic effect and contributes to autophagic regulation in cancer cells^59^. However, how autophagy is related to the PI3K–AKT–mTOR pathway is not clear. Activated mTORC1 downregulates autophagy by phosphorylating a complex of autophagy proteins (unc-51-like autophagy activating kinase 1 [ULK1]/ULK2), which inhibits the downstream autophagy cascade^60^. Furthermore, Akt can be activated via phosphorylation by mTORC2, providing positive feedback that contributes to the inhibition of autophagy^61^. In addition, active Akt can directly regulate transcription factors FOXOs (forkhead box O proteins), resulting in the inhibition of autophagy^62^.

The relationship between FGF21 and the PI3K–Akt–mTOR pathway has been reported, but the results are not consistent^63–65^. Minard et al.^63^ reported that FGF21 activated mTORC1 via MAPK rather than through the canonical PI3K–Akt pathway. Further, FGF21 activates PI3K–Akt signaling, and in turn, mTOR^66^. On the contrary, Gong et al.^65^ found that FGF21 inhibited mTORC1 activity induced by nutrients and hormones. Therefore, the relationship between FGF21 and the mTOR signal remains unclear. Herein, KEGG analysis showed that FGF21 is related to the PI3K–Akt–mTOR signaling pathway. Therefore, we speculate that FGF21 promotes autophagy in LNCaP cells by modulating PI3K-Akt–mTOR signaling. Consistent with this hypothesis, we observed that p-*FGF21* treatment downregulated the phosphorylation levels of Akt, mTOR, and their downstream target molecule, P70S6K, in LNCaP cells, indicating that PI3K–Akt–mTOR signaling was inhibited. However, mTOR agonist treatment blocked the effects of FGF21 on the conversion of LC3-I to LC3-II, and on p62, Ki-67, and BCL-2 protein expression. These data reveal that the PI3K–Akt–mTOR–p70S6K pathway plays a pivotal role in FGF21-induced autophagy and in regulating LNCaP cell proliferation and apoptosis. Indeed, an imbalance in the PI3K–Akt–mTOR pathway is a common mechanism leading to the occurrence and development of tumors^67^. Accordingly, our results present a possible mechanism for the role of FGF21 in PCa by modulating the PI3K–Akt–mTOR pathway.

As a secretory protein, FGF21 is mainly expressed and secreted in the liver under physiological conditions, but it is also expressed and secreted in other tissues under pathological conditions^33,68^. FGF21 can play a role in various tissues and organs of the human body through the circulatory system. Therefore, we believe that increasing FGF21 expression and secretion in PCa tissue or increasing circulating FGF21 level will have an important impact on the proliferation and apoptosis of PCa cells, and may become a new target for PCa treatment.

The limitations of this study are: (1) we could not determine the direct biological effects of FGF21, as there are differences between in vitro experiments and the human internal environment; (2) the effect of endogenous FGF21 on PCa remains unclear and requires further study; (3) KEGG analysis of the signaling pathway was performed on mouse adipose tissue, which may be different from prostate tissue. Moreover, we did not measure the circulating FGF21 levels in patients with PCa. Therefore, further studies are needed to determine whether FGF21 is a biomarker in patients with PCa. Nonetheless, one paper cannot solve all of the scientific problems. Despite these limitations, ours is the first study to discover the role of FGF21 in the proliferation, invasion, and apoptosis of PCa cells, and to suggest that FGF21 may be a molecular target for PCa therapy.

In summary, FGF21 is downregulated in PCa tissues and cell lines and can inhibit PCa cell proliferation and facilitate their apoptosis. GO and KEGG analyses showed that FGF21 might be related to the PI3K–Akt–mTOR signaling pathway and autophagy. Molecular biology experiments confirmed that FGF21 promoted PCa cell apoptosis through autophagy mediated by PI3K–Akt–mTOR signaling (Fig. 6E). Therefore, our results indicate that FGF21 is a potential new target for PCa therapy.

## Supplementary information

SUPPLEMENTAL TABLES

## Data Availability

All data included in this study are available and include, where applicable, accession codes, other unique identifiers and associated web links for publicly available datasets, and any conditions for access of non-publicly available datasets.
