# Supplementary material for: FGF21 facilitates autophagy in prostate cancer cells by inhibiting the PI3K–Akt–mTOR signaling pathway
Source: Cell Death Dis. 2021 Mar 22;12(4):303. doi: 10.1038/s41419-021-03588-w (PMC7985321; doi:10.1038/s41419-021-03588-w)
Supplement: Supplementary file 1 — SUPPLEMENTAL TABLES [file 41419_2021_3588_MOESM1_ESM.docx]

**Table S1** The primer sequence used for RT-PCR analysis

| Species | | Primer | Sequence (5′-3′) | Sequence length | Gene ID | Nucleotide Accession Number |
| --- | --- | --- | --- | --- | --- | --- |
| Homo | β-Actin-F | | GAGAAAATCTGGCACCACACCT | 172bp | 60 | NM_001101.5 |
|  | β-Actin-R | | GCACAGCCTGGATAGCAACGTA |  |  |  |
| Homo | FGF21-F | | CTGTGCTGGCTGGTCTTCTGC | 375bp | 26291 | NM_019113.4 |
|  | FGF21-R | | CGTGGGCTTCGGACTGGTAAAC |  |  |  |
| Homo | LC3B-F | | GATGTCCGACTTATTCGAGAGC | 167bp | 81631 | NM_022818.5 |
|  | LC3B-R | | TTGAGCTGTAAGCGCCTTCTA |  |  |  |
| Homo | P62-F | | GCACCCCAATGTGATCTGC | 92bp | 8878 | NM_003900.5 |
|  | P62-R | | CGCTACACAAGTCGTAGTCTGG |  |  |  |
| Homo | Ki67-F | | AGAAGGCAACACTACTACAACA | 198bp | 17345 | NM_002417.5 |
|  | Ki67-R | | GTTCTGTATCAGGCAAGCTCT |  |  |  |
| Homo | BCL2-F | | CTCGTCCAAGAATGCAAAGCAC | 170bp | 596 | NM_000633.3 |
|  | BCL2-R | | TCTCCCGGTTATCGTACCCTG |  |  |  |

**Table S2** Association of FGF21 with pathological features in patients with PCa

| **Characteristics N** |  | **FGF21 expression** | | ***p*-value** |  |
| --- | --- | --- | --- | --- | --- |
|  |  | **Positive (%)** | **Negative (%)** |  |  |
| **Patients** 42 |  | 26 (61.9) | 16 (38.1) |  | |
| **Age (year)** |  |  |  |  |  |
| ≤ 60 18 |  | 11 (61.1) | 7 (38.9) | 0.927 |  |
| > 60 24 |  | 15 (62.5) | 9 (37.5) |  |  |
| **PSA (ng/mL)** |  |  |  |  |  |
| ≤ 20 20 |  | 11 (55.0) | 9 (45.0) | 0.380 |  |
| > 20 22 |  | 15 (68.2) | 7 (31.8) |  |  |
| **Pathological stage** |  |  |  |  |  |
| Ta-T1 17 |  | 7 (41.2) | 10 (58.8) | < 0.05 |  |
| T2-T4 25 |  | 19(76.0) | 6 (24.0) |  |  |
| **Gleason** |  |  |  |  |  |
| G ≤ 7 18 |  | 7 (38.9) | 11 (61.1) | < 0.01 |  |
| G > 7 24 |  | 19 (79.2) | 5 (20.8) |  |  |
| **Lymph node metastasis** |  |  |  |  |  |
| No 27 |  | 18 (66.7) | 9 (33.3) | 0.394 |  |
| Yes 15 |  | 8 (53.3) | 7 (46.7) |  |  |

**Table S3** Gene ontology enrichment analysis of differentially expressed gene

| GO analysis | ID | Term | Gene count | Bg Ratio | *p* value |
| --- | --- | --- | --- | --- | --- |
| GOTERM_BP_FAT | GO:0035458 | cellular response to interferon-beta | 2 | 46/23174 | 0.000171712 |
| GOTERM_BP_FAT | GO:0035456 | response to interferon-beta | 2 | 56/23174 | 0.000254907 |
| GOTERM_BP_FAT | GO:0120162 | positive regulation of cold-induced thermogenesis | 2 | 96/23174 | 0.000747869 |
| GOTERM_CC_FAT | GO:0020003 | symbiont-containing vacuole | 1 | 14/23407 | 0.005966188 |
| GOTERM_CC_FAT | GO:0065010 | extracellular membrane-bounded organelle | 1 | 16/23407 | 0.006815881 |
| GOTERM_CC_FAT | GO:0030430 | host cell cytoplasm | 1 | 17/23407 | 0.007240481 |
| GOTERM_CC_FAT | GO:0033655 | host cell cytoplasm part | 1 | 17/23407 | 0.007240481 |
| GOTERM_CC_FAT | GO:0031091 | platelet alpha granule | 1 | 19/23407 | 0.008089193 |
| GOTERM_CC_FAT | GO:0033646 | host intracellular part | 1 | 20/23407 | 0.008513304 |
| GOTERM_CC_FAT | GO:0043656 | intracellular region of host | 1 | 20/23407 | 0.008513304 |
| GOTERM_CC_FAT | GO:0033643 | host cell part | 1 | 22/23407 | 0.009361037 |
| GOTERM_CC_FAT | GO:0018995 | host | 1 | 24/23407 | 0.010208117 |
| GOTERM_CC_FAT | GO:0043657 | host cell | 1 | 24/23407 | 0.010208117 |
| GOTERM_CC_FAT | GO:0044215 | other organism | 1 | 30/23407 | 0.012745447 |
| GOTERM_CC_FAT | GO:0044216 | other organism cell | 1 | 30/23407 | 0.012745447 |
| GOTERM_CC_FAT | GO:0044217 | other organism part | 1 | 30/23407 | 0.012745447 |

**Table S4** KEGG enrichment analysis of differentially expressed gene

| Pathway ID | Pathway name | Gene count | BgRatio | *p* value | Genes |
| --- | --- | --- | --- | --- | --- |
| mmu00790 | Folate biosynthesis | 1 | 26/8218 | 0.01572295 | Akr1b7 |
| mmu04151 | PI3K-Akt-mTOR signaling pathway | 2 | 356/8218 | 0.01715400 | Fgf21/Thbs2 |
| mmu00052 | Galactose metabolism | 1 | 32/8218 | 0.01932308 | Akr1b7 |
| mmu00040 | Pentose and glucuronate interconversions | 1 | 34/8218 | 0.02052078 | Akr1b7 |
| mmu00051 | Fructose and mannose metabolism | 1 | 35/8218 | 0.0211192 | Akr1b7 |
